# Risk Assessment Model for Complications in Minimally Invasive Hysterectomy: A Pilot Study

**DOI:** 10.3390/ijerph20010234

**Published:** 2022-12-23

**Authors:** Matteo Bruno, Francesco Legge, Cosimo Gentile, Vito Carone, Guglielmo Stabile, Federico Di Leo, Manuela Ludovisi, Christian Di Florio, Maurizio Guido

**Affiliations:** 1 Obstetrics and Gynaecology Unit, San Salvatore Hospital, 67100 L’Aquila, Italy; 2 Gynecologic Oncology Unit, Department of Obstetrics and Gynecology “F. Miulli” General Regional Hospital, Acquaviva delle Fonti, 70021 Bari, Italy; 3 Institute for Maternal and Child Health-IRCCS “Burlo Garofolo”, Department of Obstetrics and Gynaecology, 34137 Trieste, Italy; 4 Department of Life, Health and Environmental Sciences, University of L’Aquila, 67100 L’Aquila, Italy

**Keywords:** complications, minimally invasive hysterectomy, risk assessment model, minimally invasive surgery

## Abstract

Objective: To estimate the rate of intra-operative and postoperative complications, and to define the risk of 30-day major postoperative complications (Clavien-Dindo > 2) according to the presence of one of 10 different variables of minimally invasive (MI) hysterectomy; and then to create a risk assessment model easily applicable in clinical practice. Methods: A single center single arm retrolective study. Data of consecutive patients who have undergone MI hysterectomy for gynaecologic disorders between May 2018 and April 2021 were analyzed. Perioperative surgical outcomes, occurrence of intra- and postoperative complications, and readmissions within 30 days from surgery were registered. Univariate and multivariable analyses were performed to determine the factors associated with major postoperative complications. Results: Over the study period, 445 patients were included in the study. The majority of patients developed a minor event, while major complications (grade III) were required in 14 patients. None of the patients showed a grade IV or V complication. Univariate analysis was performed on patients who had developed intra- or postoperative complications from those who did not experience complications. Body mass index (BMI) (*p*-value 0.045) and surgeon’s experience (*p*-value 0.015) were found to be associated with a different surgery time. Regarding major postoperative complications, a statistically significant association was found for the variables: BMI (*p*-value 0.006), previous abdominal surgery (*p*-value 0.015), and surgeon’s experience (*p*-value 0.035) in the univariate analysis. Also in the multivariate analysis, the risk of major postoperative complications was higher in these three different variables. BMI, previous surgery, and surgeon’s experience were inserted in a reproducible risk assessment model in order to stratify the risk of major postoperative complications. Conclusions: We proposed a risk assessment model including factors not previously considered in the literature: the standardization of the surgical technique, the surgeon’s experience, the best MI approach (laparoscopy or robot-assisted), and previous abdominal surgery are crucial tools to consider. Further prospective studies with a larger population sample are needed to validate these preliminary evaluations for patients undergoing MI hysterectomy.

## 1. Introduction

Hysterectomy represents the most common non-obstetrics surgical procedure; according to the CDC report (Center for Disease Control) in the USA, each year about 400.000 hysterectomies are performed [1,2,3]. In Italy, each year more than 50.000 admissions for hysterectomy are carried out [4]. Hysterectomy is indicated for malignant genital tract diseases and for benign disorders for which medical treatment is impossible or has proven to be ineffective or is refused by the patient [5,6].

Compared to the laparotomic approach, minimally invasive surgery (MIS) is associated with lower pain intensity, blood loss, and shorter hospitalization [7,8,9,10]. However, when compared to the vaginal approach, the benefits of MIS are less clear; indeed, the American College of Obstetricians and Gynecologists [10,11,12] and recent studies [10,11,12,13,14] recommend the vaginal route as the first choice for benign disease. However, even if the vaginal approach has been considered the standard of care worldwide, most hysterectomies are still performed by MIS.

Today, indications for minimally invasive (MI) hysterectomy include malignant genital tract diseases and benign conditions for which medical treatment is ineffective such as large fibroids, pelvic prolapse, and pelvic or ovarian endometriosis [15,16,17].

The risk of a complication is dependent on patient characteristics, comorbidities, and surgeons’ expertise. Several studies have reported that surgical-related complications in MI hysterectomy procedures varies from 8.8% to 11% [18,19]; however, few studies specifically analyzed factors related to the procedure. Identification of specific factors for intra- and postoperative complications is actually a topic of discussion in the literature [17,20,21,22] and the most recent studies have evaluated the real predictive risk factors to be considered in the MI hysterectomy procedure [17,20,21]. Risk assessment models to predict postoperative morbidity and mortality have been developed in general surgery [23,24], bariatric surgery [25], and gynecology [17,20,21,22], in order to identify the risk factors that could reduce the potential for complications. Recently, Schmidt et al. [20] developed a nomogram as a tool for predicting the postoperative complications in gynecology using any surgical approach (vaginal, MIS, laparotomy). Data currently available in the literature report that sepsis, abdominal approach, diabetes, gynecological cancer, blood transfusions, American Society of Anesthesiologists Physical Status Classification System (ASA) class ≥ 3, and body mass index ≥ 40, were found to be the preoperative independent risk factors for major postoperative complications. Risk assessment factors in MI gynecological procedures are heterogeneous; however, the need for conversion to open surgery and the occurrence of any intraoperative complications were commonly found as independent factors associated with postoperative complications in MI hysterectomy [17,20,21,22].

In the present study, we analyzed data from 445 patients that underwent MI hysterectomy between May 2018 and April 2021 in a tertiary MI gynecologic center. For instance, we evaluated the risk of complications in a large single institutional series of MI hysterectomies and we analyzed potential risk factors for severe complications. Furthermore, starting from the relationship among 10 variables—including clinical parameters of the patient, surgeon’s experience, and surgical procedures–and the observed intra-operative and postoperative complications in the univariate and multivariate analyses, we developed a risk assessment model of severe complications, easily applicable in clinical practice.

## 2. Materials and Methods

### 2.1. Patients Selection

This is a retrolective single institutional study designed to evaluate the association between specific risk factors and MI hysterectomy. We performed a collection and analysis of 445 patients’ data that underwent MI hysterectomy at the Regional General Hospital “F. Miulli” in Acquaviva delle Fonti (Ba) between May 2018 and April 2021: 62 were performed in 2018, 161 in 2019, 153 in 2020, and 69 in 2021. All patients had already provided written informed consent for their data to be collected and analyzed for scientific purposes. The study was reviewed and approved by our institutional board (ID 3430). 

Patients who had surgery for benign (endometriosis, myomatous uterus, abnormal uterine metrorrhagia), premalignant (cervical dysplasias, endometrial hyperplasia), or malignant (borderline ovarian tumors, endometrial cancer, or first stage ovarian cancer) indications were included in the study. We excluded patients who had a supra-cervical or radical hysterectomy or had a preoperative diagnosis of cervical cancer due to the choice of surgical approach, the radicality of the surgical procedure, and the different rate of related complications. 

A uniform surgical approach of MI hysterectomy was adopted during the study period. A step-by-step description of the surgical technique is provided elsewhere [17,26]. A hysterectomy is started with the coagulation and section of the round ligament. The broad ligament is opened up to the utero-vesical fold that is then incised with caudal reflection of the bladder. The uterine arteries are closed by clips at the origin. The ovaries are left in place or removed based on the patient’s age and indication for surgery, while the fallopian tubes are always removed. Once the uterine vessels are coagulated and transected, the hysterectomy is completed with a monopolar hook. The uterus is then extracted from the vagina and a vaginal morcellation in endobag is accomplished, when required. The suture of the vaginal cuff is performed laparoscopically. 

Variables initially considered as potential risk factors for the occurrence of procedure-related complications were: primary diagnosis (benign or premalignant/malignant disease), patient’s age, body mass index (BMI), presence of diabetes or hypertension, ASA risk class, previous history of abdominal surgery, further surgical procedures other than MI hysterectomy (omentectomy, pelvic sentinel lymph node removal, peritoneal biopsies, adhesiolysis, pelvic or lombo-aortic lymphadenectomy), surgeon’s experience, and the surgical technique. A total of 366 were performed by standard laparoscopy while 79 patients (17.8%) underwent robot-assisted surgery (Da Vinci Xi^®^).

Obesity was defined as a BMI ≥ 30 kg/m^2^, according to the World Health Organization classification [27]. Considering the median age as 51 years old, we considered this value as the cut off. Surgeon’s experience was analyzed in two categories: the first was designated ‘‘novel period’’ and comprised data for the first 75 MI hysterectomies done by each surgeon. The second, designated ‘‘routine period’’, was used for data from the subsequent 75 MI hysterectomies. This classification was developed on the basis of earlier findings [27,28,29,30,31] that between 50 and 100 laparoscopic hysterectomy (LH) are needed for surgeons to surmount the learning curve satisfactorily. 

The perioperative-evaluated outcomes were retrieved from medical records: operative time (min), estimated blood loss (EBL) (mL), need for conversion to laparotomy, and days of hospital stay. Surgical complications were categorized according to the time of appearance as intraoperative or postoperative, and according to the grade of severity using the Clavien–Dindo classification [32]. The Clavien–Dindo classification defines a complication as any deviation from the ideal postoperative course that is not inherent to the operation and that cannot be considered as a therapeutic failure of the operation. In concept, the classification is made according to the grade of severity based on the respective therapeutic intervention that led to treatment of the observed deviation [32]. Postoperative complications within 30 days from surgery were divided into minor complications (grade I and II) and major complications (grade III, IV and V). 

### 2.2. Statistical Analysis

The sample has been described in its clinical and demographic characteristics, applying the descriptive statistic technique. Descriptive statistics for continuous and categorical variables have been reported as an absolute number (mean and standard deviation or median and range), a rate, and a percentage (%). A comparison of the demographic and surgical characteristics with the occurrence of complications (according to the Clavien-Dindo classification) have been performed using the Chi-square test or Fisher’s exact test. Continuous data comparisons have been performed with the Wilcoxon signed rank test. Statistical tests used to calculate the *p*-values were two-tailed; all *p*-values < 0.05 were considered statistically significant. Statistical analysis has been performed with SPSS v. 21.0 software. 

## 3. Results

A total of 445 patients underwent a MI hysterectomy at our institution over the three year study period and baseline characteristics have been reported in Table 1; of these, 63.4% of hysterectomies (*n* = 282) were performed for benign disease while 36.6% (163 cases) were performed for premalignant or malignant disease. The median age was 51 (±0.26) years. Patients were classified as normal BMI (295 cases, 66.3%) and obese BMI (150 cases, 33.7%); mean BMI was 24.8 (±0.32). The presence of diabetes or hypertension and ASA class are shown in Table 1. A history of previous abdominal surgery, either laparoscopy or laparotomy, was evaluated; a total of 142 patients (31.9%) were found had previous surgery.

Additional procedures were carried out in 304 cases (68.3%) while in the remaining 141 cases (31.7%), the only procedure performed was a MI hysterectomy with bilateral salpingectomy and with or without oophorectomy. Finally, 319 surgeries (71.7%) were performed by highly experienced surgeons in the “routine period”, while the remaining 126 surgeries (28.3%) were performed by surgeons in the “novel period”.

An analysis of the surgical-related parameters showed a mean operative time for standard laparoscopy hysterectomies of 145 (±8.7) minutes, and the mean time for robot-assisted surgery of 215 (±11.3) minutes. The mean estimated blood loss was 100 (±35.5) mL. The mean length of hospital stay was 4.8 (±0.2) days. 

The percentage of intraoperative and postoperative complications according to the Clavien–Dindo classification was the following: 44 patients (9.88%) showed a related surgical complication and among these, eight occurred intraoperatively and 36 were described in the 30-day postoperative course. Among the eight intra-operative complications, conversion to open surgery occurred in three cases as a result of the considerable size of the uterus and of the presence of tenacious post-surgery adhesions; two patients developed bladder injuries during the detachment of the vesical-uterine fold; in one case there was a vaginal mucosa lesion that occurred during the extraction of the uterine body; in one case there was a rectum lesion (due to the presence of an endometriosis node); and finally in one case the patient suffered an injury of the obturator nerve (interruption of 70% of the obturator nerve caliber), which was repaired intraoperatively. 

Details of the post-operative complications are reported in Table 2. The majority of patients developed a minor event, while complications requiring surgical, endoscopic, or radiological intervention (grade III) were required in 14 patients. Three patients developed a ureteral fistula and were treated with a double J ureteral stent while one patient had a ureteral stenosis requiring reoperation with a laparoscopic ureteroneocystostomy. Three patients developed a vaginal cuff dehiscence and a new surgery was required. None of the patients showed a grade IV or V complication.

For the univariate analysis, patients were compared for mean surgery time and for mean EBL using the clinical-pathological characteristics described. A univariate analysis was performed on patients who had developed intra- or postoperative complications from those who did not experience complications. As shown in Table 3, BMI (*p*-value 0.045) and surgeon’s experience (*p*-value 0.015) were found to be associated with a different surgery time. No one clinic-pathological characteristic was found to be correlated with mean EBL or with intra-operative complications. 

The analysis of variables as risk factors, in correlation to minor postoperative complications, showed a statistically significant association (*p*-value< 0.05) for three of 10 variables: type of disease (*p*-value 0.015), age (*p*-value 0.015), and BMI (*p*-value 0.003) (Table 4).

Instead, regarding major postoperative complications (grade III), a statistically significant association was found for the variables: BMI (*p*-value 0.006), previous surgery (*p*-value 0.015), and surgeon’s experience (*p*-value 0.035) (Table 4). 

Table 5 summarizes the odds ratio (OR) obtained with logistic regression models to estimate the effect of surgeon’s experience, BMI, and previous surgery on different post-MI hysterectomy complications with adjustment for age and type of disease (benign or malignant). The risk of postoperative grade III complications was higher in the three statistically significant variables.

The three significant variables defined as possible risk factors correlated to MI hysterectomy severe complications were inserted in a risk assessment model to stratify the risk of postoperative severe complications.

A 0–1 value was assigned to each of the three variables and adding the value of each single variable obtained a score from 0 to 3. 

As shown in Table 6, the application of a score from 0 to 3 to the 445 patients analyzed showed that:

- score 0 was present in 148 patients and among these, only one reported severe complications (0.67%);
- score 1 was present in 200 patients and among these, two reported severe complications (1.05%);
- score 2 was present in 82 patients and among these, seven showed adverse events (8.5%); and
- score 3 was present in 15 patients and among these, four showed complications (26.7%).

Using this risk assessment model, it is possible to stratify the risk of severe postoperative complications according to the presence of the analyzed variables.

## 4. Discussion

The most recent Cochrane review recommends the vaginal route for hysterectomy, if possible, in consideration of the evidence reporting a higher complication rate in the MI approach [10,11,12,33]. However, some researchers have expressed disagreement, noting that this increased risk is related to a lack of adequate patient selection and a reduced experience in MI surgery [34,35,36]. In this retrolective analysis of MI hysterectomies, we have collected complications related to the procedure and we have researched the independent risk factors of major and minor postoperative events in order to create a score that can allow them to be considered in the routine of clinical practice.

Literature data confirm that minimally invasive hysterectomy is a safe and effective approach and at the same time, in our analysis we highlight that performing these procedures in a referenced tertiary center and standardizing the surgical technique reduces the risks related to the procedure.

Overall, we found an acceptable postoperative complications rate with a low risk of severe events; major postoperative complications (grade III) were recorded in 3.1% of cases, while the total complications amounted to around 8% of the procedures performed. It should be noted that MI surgery is a surgical approach with infrequent severe complications; this is shown by our results: we do not have type IV and type V complications. 

Reports indicate that the incidence of ureteral injury is estimated to be low, although some reports of MI hysterectomy highlight a higher risk or ureteral lesions compared with the vaginal approach [37,38]; our ureteral complication rate is low, about 0.8%. One randomized trial that involved more than 1.300 hysterectomies reported a bladder injury rate of 2.1% for the laparoscopic route and 1.25% for the vaginal route [39] while other trials reported ranges from 0.2% to 8.3% for a laparoscopic hysterectomy. From this study, there was a very low risk of bladder injury during MI hysterectomy (0.3%) and this risk is greater than during a vaginal hysterectomy.

Regarding the incidence of vaginal cuff dehiscence [26,40,41], this ranged in the literature between 0.64 and 1.35% [26,42]; our rate is 0.435%. A multi-institutional analysis by Uccella et al. [26] revealed that laparoscopic closure of the vaginal cuff at the end of a total laparoscopic hysterectomy is associated with a significant reduction in vaginal dehiscence and any cuff complication. Meanwhile, Clarke–Pearson and Geller [43] reported a meta-analysis that discovered that the robotic closure has a higher risk of vaginal cuff dehiscence compared to the laparoscopic and vaginal one. Our low rate of vaginal cuff dehiscence is due to the systematic use of the laparoscopic closure as suggested by the randomized clinical trial of Uccella et al. [26] and likely due to the systematic administration of a vaginal suppository after surgery. However, there are no studies that have specifically assigned a role of training in laparoscopic suturing, although we believe that the surgeon’s ability in the closure technique is closely related to this type of complication. 

Recent works by Danish groups reported that MI hysterectomy (laparoscopy LPS and robot-assisted LPS) for a benign condition is associated with a lower risk of major complications within 30 days of surgery compared to the vaginal approach [44,45].

Our analysis highlights BMI, previous surgery, and surgeon’s experience as independent risk factors for major postoperative complications, as reported by other authors [46,47]. 

Regarding the Clavien–Dindo classification of complications, we observed that as the surgeon’s experience decrease, type III complications increase; the most recent meta-analysis [30,48] showed that virtual reality training in laparoscopic surgery improves the efficiency and quality by reducing error rates. Naveiro-Fuentes et al. [27] demonstrated that the surgeon’s experience in laparoscopic hysterectomy plays an essential role in decreasing the complication rate. Therefore, we believe that the effect of the operator’s experience is an important factor in reducing complications in MI surgery and it should always be considered according to the type of procedure. Surgeons with greater skills in MI surgery should be privileged in conditions such as pre-malignant pathology, endometriosis, or previous surgery.

BMI is a recognized risk factor in MI surgery procedures [49,50]. In gynecology, for malignant or premalignant conditions, a total hysterectomy with bilateral salpingo-oophorectomy with or without lymph node sentinel biopsy or lymphadenectomy is standard care. For this indication, robot-assisted laparoscopy has been shown to be superior to the laparotomic approach, [49,50,51,52] and shows comparable or slightly beneficial results in terms of shorter length of stay. The subgroup analysis of our patients undergoing robotic surgery showed that BMI is not a risk factor, as reported in the literature [49,50,51]. We believe that given the increased risk of complications related to a higher BMI and MI surgery, the robotic approach should be considered routinely in clinical practice [53].

We created a risk assessment model to assess the risk of complications within 30 days of a MI hysterectomy. This analysis should be compared to other risk models present in the literature: Erekson et al., [21] as well as Casarini et al., [17,44,45] evaluated the complications after a hysterectomy for benign conditions while we included a premalignant and malignant pathology. Second, we included women who had undergone a hysterectomy using only the MI approach, different from other authors such as Schmidt [20] or Hiesler [22]. Third, our score has an important strength: it is the first risk assessment model that considers the importance of factors dependent on the surgeon, such as experience and skills in MIS, or factors related to previous surgery (e.g., adherential syndrome or previous retroperitoneal surgery). Finally, our model could represent a simple model–it includes only three variables–and could be reproduced in any hospital. 

We performed a sample size calculation based on the 10 variables and major complications according to the Simon two-stage design, using an alpha error of 0.05 and a beta error of 0.80 for each variable. The sample size for the medical variables evaluated (e.g., diabetes, hypertension, and age) is stronger to obtain than the MI procedure-related variables (e.g., 320 women for the surgical technique and 486 women for the surgeon’s experience). Since we set our analysis as a pilot study, this sample size can be useful for planning for future prospective studies, including specific risk factors related to MI surgery. Hypertension and diabetes are currently controlled pathologies, as well as age and ASA risk class, and have been extensively analyzed in MI surgery. Therefore, future research will mainly focus on those specific MI risk factors, such as the experience of the surgeon in MI hysterectomy or the type of MI technique used, including sample size calculations based upon the expected risk of severe complications in the different study subgroups.

We believe that the application of the risk assessment model in a prospective analysis can impact the correct clinical approach towards the patient undergoing a MI hysterectomy. Further prospective studies are needed to validate these preliminary evaluations. The external validation of the score would guarantee a reduction in the limits related to this study. A limitation of our study was the retrolective nature of the study. Another limitation is represented by the sample size population in which the subgroup analysis was carried out (e.g., major postoperative complications). This is evident in the low degree of precision of the confidence intervals for the variables BMI, previous surgery, and surgeon’s experience. 

As many patients undergo surgery for MI hysterectomy, information regarding the extent of its benefits can be useful for decision-making as well as for referrals to expert centers able and willing to perform surgery to achieve minor complication rates.

## 5. Conclusions

The standardization of the surgical technique, the surgeon’s experience, and the best MI approach (LPS or robot-assisted) are fundamental tools to consider. This knowledge is important to personalize the treatment for patients before undergoing MI hysterectomy and to minimize the risk of major postoperative complications. Prospective studies with a larger population sample could validate and confirm this risk assessment model as a tool to be used routinely in women undergoing MIS for a benign or malignant hysterectomy.

## Figures and Tables

**Table 1:** Baseline characteristics of the study population.

Variables and Demographic Data	N = 445 (%)
**Primary diagnosis**	
Benign	282 (63.4%)
Malignant	163 (36.6%)
**Age, years at surgery**	
Mean	51 (±0.26)
<51 yo	229 (51.5%)
≥51 yo	216 (48.5%)
**BMI**	
Mean	24.8 (±0.32)
Normal	295 (66.3%)
Obese	150 (33.7%)
**Diabetes**	
Yes	32 (7.2%)
No	413 (92.8%)
**Hypertension**	
Yes	119 (26.8%)
No	326 (73.2%)
**ASA risk class**	
I	94 (21.1%)
II-III	351 (78.9%)
**Previous abdominal surgery**	
Yes	303 (68.1%)
No	142 (31.9%)
**Additional procedures**	
Yes	141 (31.7%)
No	304 (68.3%)
**Surgical technique**	
Standard laparoscopy	366 (82.2%)
Robot-assisted	79 (17.8%)
**Surgeon’s experience**	
Routine period	319 (71.7%)
Novel period	126 (28.3%)

**Table 2:** Details of postoperative complications using Clavien–Dindo classification.

Degree (N)	Type of Complication (N)
**I (11)**	Vaginal cuff bleeding (8)
	Paralytic ileus (3)
**II (11)**	Fever (3)
	Vaginal cuff dehiscence without reintervention (1)
	Urinary retention (1)
	Recurrent cystitis (2)
	Blood transfusion (4)
**III (14)**	Vaginal cuff dehiscence with reintervention (3)
	Abdominal hernia (2)
	Hemoperitoneum (3)
	Ureteral fistula (3)
	Ureteral stenosis (1)
	Bowel injury (1)
	Suprafascial hematoma (1)

**Table 3:** Univariate analysis of intraoperative parameters and complications based on clinical-pathological characteristics.

Characteristics	Mean Surgery Time (min)	*p*-Value ^a^	Mean EBL (mL)	*p*-Value ^a^	Intraoperative Complications	*p*-Value ^a^
**Primary diagnosis**						
Benign	140	0.207	80	0.693	5	0.897
Malignant	155		95		3	
**Age, years at surgery**						
<51 yo	150		100		4	0.628
≥51 yo	170	0.345	100	0.881	4	
**BMI**						
Normal	145	0.045	100	0.367	4	0.190
Obese	180		100		4	
**Diabetes**					7	…
Yes	135	0.673	100	0.533	1	0.612
No	130		93			
**Hypertension**						
Yes	155	0.569	100	0.213	6	0.701
No	150		100		2	
**ASA risk class**						
I	145	0.967	110	0.873	2	0.855
II-III	150		100		6	
**Previous**						
**abdominal**						
**surgery**						
Yes	150	0.614	115	0.611	5	0.894
No	170		100		3	
**Additional**						
**procedures**						
Yes	145	0.146	110	0.604	3	0.387
No	160		100		5	
**Surgical**						
**technique**						
Standard LPS	145	0.073	100	0.827	7	0.597
Robot-assisted	215		60		1	
**Surgeon’s**						
**experience**						
Routine period	130	0.015	100	0.665	6	0.889
Novel period	160		70		2	

^a^ All comparisons were made by two-tailed *t*-test statistics.

**Table 4:** Univariate analysis of postoperative complications based on clinical-pathological characteristics.

Characteristics	Minor Postoperative Complications N.	*p*-Value ^a^	Major Postoperative Complications N.	*p*-Value ^a^
**Primary diagnosis**				
Benign	12	0.015	10	0.607
Malignant	10		4	
**Age, years at surgery**				
<51 yo	15		7	0.673
≥51 yo	7	0.013	7	
**BMI**				
Normal	6	0.003	3	0.006
Obese	16		11	
**Diabetes**				
Yes	21	0.482	13	0.929
No	1		1	
**Hypertension**				
Yes	12	0.164	11	0.215
No	10			1
**ASA risk class**	5	0.829	0	0.994
I	17		14	
II-III				
**Previous abdominal**				
**surgery**				
Yes	16	0.624	5	0.015
No	6		9	
**Additional procedures**				
Yes	5	0.876	2	0.825
No	17		12	
**Surgical technique**				
Standard LPS	19	0.234	14	0.995
Robot-assisted	3		0	
**Surgeon’s experience**				
Routine period	16	0.765	6	0.035
Novel period	6		8	

^a^ All comparison were made by two-tailed *t*-test statistics.

**Table 5:** Predictors of major (grade III) complications following MI hysterectomy: multivariable analysis.

	Odds Ratio	Lower 95% CI	Upper 95% CI	*p*-Value ^a^
Primary diagnosis	2.65	0.80	8.30	0.11
Age	1.55	1.40	8.35	0.25
BMI	3.66	1.10	16.50	<0.001
Previous surgery	4.10	1.35	13.89	0.02
Surgeon’s experience	3.77	1.25	11.98	0.033

^a^ All comparisons were made by two-tailed *t*-test statistics.

**Table 6:** Risk assessment model.

Score	Patients (N)	Severe Postoperative Complications (%)
**0**	148	1 (0.67%)
**1**	200	2 (0.95%)
**2**	82	7 (8.5%)
**3**	15	4 (26.7%)

## Data Availability

The authors confirm that the data supporting the findings of this study are available within the article.
